# Exposure to *Leishmania* spp. infection and *Lutzomyia* spp. in individuals living in an area endemic for visceral leishmaniasis in Brazil

**DOI:** 10.1590/0037-8682-0320-2019

**Published:** 2019-12-20

**Authors:** Karina Yukie Hirata, Edenilson Borges de Oliveira, Lais Rigon, Yuri Tani Utsunomiya, Thaise Yumie Tomokane, Márcia Dalastra Laurenti, Mary Marcondes

**Affiliations:** 1 Universidade Estadual Paulista, Faculdade de Medicina Veterinária de Araçatuba, Programa de Pós-Graduação em Ciência Animal, Araçatuba, SP, Brasil.; 2 Universidade Estadual do Norte do Paraná, Faculdade de Medicina Veterinária, Centro de Ciências Agrárias, Bandeirantes, PR, Brasil.; 3 Universidade Estadual Paulista, Faculdade de Medicina Veterinária de Araçatuba, Departamento de Apoio, Produção e Saúde Animal, Araçatuba, SP, Brasil.; 4 Universidade de São Paulo, Faculdade de Medicina, Departamento de Patologia, São Paulo, SP, Brasil.; 5 Universidade Estadual Paulista, Faculdade de Medicina Veterinária de Araçatuba, Departamento de Clínica, Cirurgia e Reprodução Animal, Araçatuba, SP, Brasil.

**Keywords:** Antibodies, Humoral immunity, Sandfly saliva, Serology

## Abstract

**INTRODUCTION:**

This study aimed to investigate human exposure to *Leishmania* spp. infection and sandflies in an area endemic for the disease.

**METHODS:**

The presence of antibodies specific for *Leishmania* spp. and saliva of *Lutzomyia* spp. and that of *L. infantum* DNA in blood were evaluated.

**RESULTS:**

Antibodies against *Leishmania* spp. and sandfly saliva were observed in 20.8% and 37.7% of individuals, respectively. DNA of *Leishmania* spp. was amplified from the blood of one patient.

**CONCLUSIONS:**

The results suggest that *Leishmania* spp. infection may be underdiagnosed in this area.

The accurate diagnosis of human visceral leishmaniasis (VL) is still challenging. VL should always be clinically suspected when a patient living in an area in which the disease is endemic presents with fever and splenomegaly, with or without hepatomegaly, and the diagnosis should be made as accurately and early as possible. However, failures in surveillance with unidentified asymptomatic cases and patients who die without confirmation of the disease contribute to the underdiagnosis of human VL in Brazil^1^. 

Sandfly saliva contains several molecules that can modulate the host immune response and influence the course of *Leishmania* spp. infection, including maxadilan, which has vasodilatory, anticoagulant, and immunosuppressive properties^2^. However, some evidence suggests that previous establishment of a specific immune response against sandfly saliva may reduce the infectivity of the pathogen and stimulate the development of a protective cellular immune response^2^. The importance of sandfly saliva in *Leishmania* spp. infection in humans has not yet been fully elucidated; however, the production of antibodies against the saliva has been evaluated in conjunction with the induction of delayed type hypersensitivity (DTH) in individuals infected with *L. infantum* to verify the role of such antibodies in the host immune response^3^. 

Due to the territorial expansion of human VL in Brazil and because the disease may be underdiagnosed in individuals living in endemic areas, the present study aimed to investigate exposure to *Leishmania* spp. infection and sandflies in individuals who were referred to a hospital located in an area endemic for the disease.

This study was approved by the Ethics Committee for Experimentation Involving Human Beings of São Paulo State University, Araçatuba (protocol CAEE: 39096314.8.0000.5420). The samples were obtained from individuals who were referred to a hospital in the micro-region of Araçatuba, composed of 16 counties, in São Paulo State, Brazil, an area with ​​intense transmission of VL and high prevalence of canine visceral leishmaniasis (CanVL). Patients who needed to undergo blood collection were invited to participate in the study. The blood aliquots were separated as follows: one for the serological tests and the other for *Leishmania* polymerase chain reaction (PCR). Individuals were eligible for the study if (a) they were aged at least 2 years; (b) they had no previous history of VL; and (c) they lived in one of the municipalities of the micro-region. Of 1,238 individuals referred to the public hospital who underwent blood collection, 284 agreed to participate in the study.

Enzyme-linked immunosorbent assay (ELISA) for *Leishmania* spp. using crude *L. infantum* antigen (MHOM/BR/72/strain46) and anti-human IgG peroxidase conjugate (Sigma-Aldrich, A6029) was performed according to the method of Laurenti et al.^4^. ELISA for *Lu. longipalpis* saliva, using as antigen salivary gland lysate (SGL) from *Lu. longipalpis* captured in Cametá municipality, Pará state, Brazil, was performed according to the method of Rohousova et al.^5^. SGL was produced according to the method of Batista et al.^6^. All samples were evaluated in triplicate. Negative and positive controls were included in each plate, and values were expressed as triplicate optical densities (ODs). Cutoff values were determined by analysis of serum samples from healthy individuals from an area non-endemic for VL. The mean value plus 3 standard deviations was considered as the cutoff point. The ODs were expressed in ELISA units (EUs). The cutoff points for anti-*Leishmania* spp. and anti-saliva were 38.51 EUs and 29.43 EUs, respectively.

Samples of whole blood were also used for *Leishmania* spp. DNA amplification by PCR, according to the method of Marcondes et al.^7^. The target *Leishmania* DNA for PCR amplification was a 116-base-pair fragment in the constant region of the kinetoplast DNA minicircle. Briefly, the reaction was performed using a commercial mastermix with SYBR Green fluorophore (SYBRGreen JumpStart TaqReadMix S4438, Sigma-Aldrich, St Louis, MO, USA), 900 nM of each primer (JW11 (forward), 5’-CCTATTTTACACCAACCCCCAGT-3’, and JW12 (reverse), 5’-GGGTAGGGGCGTTCTGCGAAA-3’), and 5 μL of DNA, in a final volume of 25 μL. Samples (tested in triplicate) were placed into 96-well PCR plates, and PCR amplification was performed in a thermocycler (CFX96TM Real-Time System, Bio-Rad, Hercules, CA, USA) using the following conditions: 94°C for 2 min and 40 cycles of 94°C for 15 s, followed by 60°C for 1 min, when fluorescence data were collected. To conduct a melting curve analysis, the temperature was increased from 60°C to 95°C, with an increment of 0.5°C every 5 s. Each amplification run contained a positive control (DNA extracted from 1.6 × 10^4^ *L. infantum* promastigotes) in triplicate to test the proper conditions of the reagents and negative controls with ultrapure water in triplicate to monitor cross-contamination^8^.

Associations between serological results and the variables, age and sex, were evaluated using Pearson’s chi-squared test for statistical independence with Yates’s correction for continuity. The significance level was adjusted for multiple testing using the Bonferroni correction, which resulted in a probability of 1% of wrongly rejecting the null hypothesis of no association. Additionally, the linear correlation between titers of antibodies specific for *Leishmania* spp. and *Lutzomyia* spp. was analyzed using Pearson’s product-moment correlation coefficient. All statistical analyses were performed using R software version 3.4.3 (R Core Team, 2018).

Based on the serology and PCR results, of 284 evaluated patients, 60 (21.1%) were considered to have been exposed to *Leishmania* spp. Data related to sex and age of the population are shown in Table 1. Antibodies against *Leishmania* spp. were observed in 59 (20.8%) patients, while antibodies against sandfly saliva were observed in 107 (37.7%). A total of 58 (20.4%) patients were seropositive in both tests, 49 (17.3%) had anti-saliva antibodies without the presence of anti-*Leishmania* spp. IgG, and one (0.4%) had anti-*Leishmania* spp. IgG without the presence of antibodies against the sandfly saliva. In 176 (61.9%) individuals, a negative result was observed for both serological tests. 

**TABLE 1:** Number and percentage, according to sex and age, of 284 patients who were referred to a public hospital in an area endemic for visceral leishmaniasis, in the micro-region of Araçatuba, SP, considered exposed to *Leishmania* spp. according to the presence of anti-*Leishmania* spp. antibodies or amplification of *Leishmania* spp. DNA fragment by real-time PCR.

Sex	Total group	Exposed group
	(n=284)	(n=60)
Male	92 (32.4%)	12 (20.0%)
Female	192 (67.6%)	48 (80.0%)
Age group		
≤18 years	9 (3.2%)	1 (1.7%)
19-65 years	226 (79.6%)	51 (85.5%)
>65 years	49 (17.3%)	8 (13.3%)

The glyceraldehyde-3-phosphate dehydrogenase (GAPDH) housekeeper gene was consistently amplified from all samples subjected to PCR. *Leishmania* spp. DNA was amplified from the blood sample in only one patient (0.35%), a man with non-VL-compatible clinical signs, whose serologies were negative for both *Leishmania* spp. and sandfly saliva. Of the patients with positive serological tests, 35.6% (21/59) had anti-*Leishmania* spp. EUs higher than twice the cutoff point, while 73.8% (79/107) had anti-saliva EUs higher than twice the cutoff point, reaching values of up to five times higher. 

A significant association was observed between the presence of anti-*Leishmania* spp. and anti-saliva antibodies (p <0.01) (Table 2). Regarding anti-*Leishmania* spp. and anti-saliva EUs, a highly significant regression was observed (p <0.05) (Figure 1), suggesting moderate to strong correlation (r=0.683). There was no significant association between the presence of anti-*Leishmania* spp. antibodies and sex (p=1.000) or age (p=0.290) and between the presence of anti-saliva antibodies and sex (p=0.826) or age (p=0.621) (Table 2).

**TABLE 2:** Associations between the presence of anti-*Leishmania* spp. and anti-saliva of *Lutzomyia* spp. antibodies, sex and age of 284 patients who were referred to a public hospital in an area endemic for visceral leishmaniasis, in the micro-region of Araçatuba, SP.

Response variable	Independent variable	Statistic (χ^2^)^a^	Degrees of freedom	P-value
Anti-*Leishmania*	Sex	0.000	1	1.000
Anti-*Leishmania*	Age	2.473	2	0.290
Anti-*Leishmania*	Anti-saliva	113.345	1	p<0.01
Anti-saliva	Sex	0.048	1	0.826
Anti-saliva	Age	0.954	2	0.621

a Pearson’s test of independence.

**FIGURE 1:**
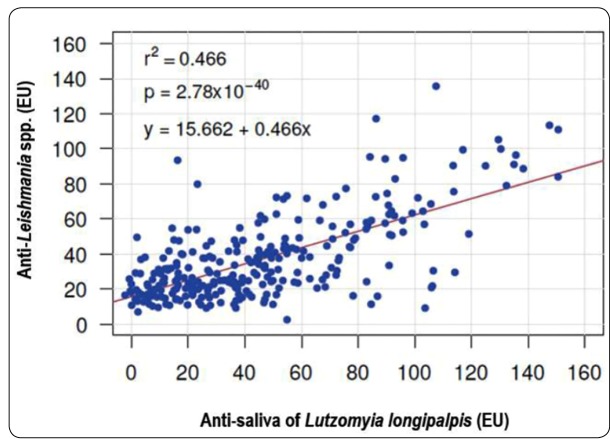
Scatter plot and adjusted regression line for anti-*Leishmania* spp. and anti-*Lutzomyia* spp. saliva antibodies (ELISA units [EUs]) from patients who were referred to a hospital in an area endemic for visceral leishmaniasis in Brazil.

The prevalence of anti-*Leishmania* spp. antibodies (20.8%) in the studied population was similar to that observed in a study conducted in the state of Rio Grande do Norte (24.6%), an area also endemic for VL in the Northeast of Brazil^9^. A positive serological test in humans may be used to confirm a recent or acute infection by *L. infantum*, regardless of the presence of symptoms, but does not indicate a risk of progression to the clinical form of the disease^10^. Generally, most individuals infected with *L. infantum* have asymptomatic infection^10^. In these cases, there is an increase in the serum antibody titer after the infection, followed by a decrease over time, while a predominantly cellular immune response develops, which can be evidenced by the presence of a DTH reaction. In contrast, individuals who progress to the clinical form of the disease have high titers of antibodies, which tend to decrease only after effective treatment^9^ ^,^ ^10^. 

The prevalence of parasitemia identified by PCR in the studied population was 0.35%, lower than the 17% observed in individuals living in neighborhoods adjacent to those with confirmed cases of VL.^9^ DNA amplification from the peripheral blood should always be interpreted together with the clinical presentation and results of other diagnostic tests^11^ and does not necessarily indicate a risk of progression of the infection. The presence of parasite DNA in the absence of serum antibodies against the parasite most likely indicates a recent infection, in which there has been insufficient time for seroconversion^9^. 

About 37.7% of the evaluated patients had antibodies against sandfly saliva. The high titers of such antibody, detected in the majority of seropositive patients, could suggest that repeated exposures to the vector occur in individuals living in endemic areas^12^. In the present study, 20.4% of patients had antibodies against both sandfly saliva and *Leishmania* spp., and there was a moderate to strong correlation between the intensities of both serological responses, varying from the result of a previous study in which children who had anti-*L. chagasi* antibodies did not develop a humoral immune response against the saliva of the vectors^3^. In the abovementioned study, individuals who did not have an immune response against salivary proteins developed anti-*Leishmania* antibodies associated with disease progression^3^. The presence of *Leishmania* spp. antibodies without the concomitant presence of antibodies against sandfly saliva was observed in only one (0.4%) patient of the present study; however, we did not have access to the information on his clinical evolution. 

In 17.3% of patients, it was possible to identify anti-saliva antibodies without anti-*Leishmania* spp. antibodies. This could be due to several factors including the possibility of being exposed to non-infected sandflies, time necessary for seroconversion after exposure to the parasite, or development of a predominantly cellular immune response in individuals who, even though infected, did not have detectable antibodies against *Leishmania* spp.^9^ ^,^ ^10^. Sandfly saliva contains several pharmacologically active substances capable of interfering with the host’s inflammatory and immunological responses. In naive hosts, co-inoculation of sandfly saliva and parasites increases the likelihood of infection^13^; however, hosts that are repeatedly exposed to sandflies and exhibit high titers of anti-saliva antibodies develop a protective immune response against *Leishmania* spp. infection^13^. This suggests that significant exposure to the bites of sandflies could be associated with the development of a protective immune response against *Leishmania*, driven by factors contained within the vector saliva^14^. 

The stimulation of a cellular immune response against the parasite after previous contact with *Lu. longipalpis* saliva may be due to a DTH reaction at the site of the bite, which may transform the lesion and its surroundings into an inhospitable site for the development of *Leishmania* infection^12^.

 Although several studies have been conducted with the same technique, using a crude *Lu. longipalpis* SGL to obtain the antigen^2^ ^,^ ^3^, it is impossible to rule out a cross-reaction between the antigens present in the SGL and those present in the saliva of other insects^2^. Moreover, some points regarding the limitation of serological tests should be considered, such as false negative results in cases of low antibody titers, reflecting the sensitivity of the test, and false positive results in the presence of co-infections, reflecting the specificity of the test. Another point that should be considered is that asymptomatic individuals, such as those evaluated in this study, generally present low circulating antibody titers, due to either low and/or recent exposure or the predominance of a cellular immune response over humoral immune response^10^, compromising the accuracy of the serological test^15^. 

It is important to highlight that approximately 20% of patients living in an area endemic for VL, who were referred to a hospital with a diagnosis of fever of unknown origin, had anti-*Leishmania* spp. antibodies, but none of them had VL as a possible diagnosis. Therefore, the results of the present study suggest that infection by *Leishmania* spp. may be underdiagnosed in the region. VL should be included in the differential diagnosis of patients referred to hospitals, particularly in endemic areas. 

## References

[1] BRASIL. Ministério da Saúde (2017). Leishmaniose visceral.

[2] Aquino DM, Caldas AJ, Miranda JC, Silva AA, Barral-Netto M, Barral A (2010). Epidemiological study of the association between anti-Lutzomyia
longipalpis saliva antibodies and development of delayed-type
hypersensitivity to Leishmania antigen. Am J Trop Med Hyg.

[3] Gomes RB, Brodskyn C, Oliveira CI, Costa J, Miranda JC, Caldas A (2002). Seroconversion against Lutzomyia longipalpis saliva concurrent
with the development of anti-Leishmania chagasi delayed-type
hypersensitivity. J Infect Dis.

[4] Laurenti MD, Leandro MV, Tomokane TY, De Lucca HRL, Aschar M, Souza CSF (2014). Comparative evaluation of the DPP® CVL rapid test for canine
serodiagnosis in area of visceral leishmaniasis. Vet Parasitol.

[5] Rohousova I, Ozensoy S, Ozbel Y, Volf P (2005). Detection of species-specific antibody response of humans and
mice bitten by sand flies. Parasitology.

[6] Batista LFS, Da Matta VLR, Tomokane TY, Pacheco AD, Silveira FT, Rossi CN (2016). Canine antibody response to Lutzomyia longipalpis saliva in
endemic area of visceral leishmaniasis. Rev Soc Bras Med Trop.

[7] Marcondes M, Hirata KY, Vides JP, Sobrinho LSV, Azevedo JS, Vieira TSWJ (2018). Infection by Mycoplasma spp., feline immunodeficiency virus and
feline leukemia virus in cats from an area endemic for visceral
leishmaniasis. Parasit Vectors.

[8] Ranasinghe S, Rogers ME, Hamilton JG, Bates PA, Maingon RD (2008). A real-time PCR assay to estimate Leishmania chagasi load inits
natural sand fly vector Lutzomyia longipalpis. Trans R Soc Trop Med Hyg.

[9] Lima ID, Queiroz JW, Lacerda HG, Queiroz PVS, Pontes NN, Barbosa JDA (2012). Leishmania infantum chagasi in Northeastern Brazil: asymptomatic
infection at the urban perimeter. Am J Trop Med Hyg.

[10] Silveira FT, Lainson R, Crescente JA, Souza AAA, Campos MB, Gomes CMC (2010). A prospective study on the dynamics of the clinical and
immunological evolution of human Leishmania (L.) infantum chagasi infection
in the Brazilian Amazon region. Trans R Soc Trop Med Hyg.

[11] Ruiter CM, Van Der Veer C, Leeflang MMG, Deborggraeve S, Lucas C, Adams ER (2014). Molecular tools for diagnosis of visceral leishmaniasis:
systematic review and meta-analysis of diagnostic test
accuracy. J Clin Microbiol.

[12] Vinhas V, Andrade BB, Paes F, Bomura A, Clarencio J, Miranda JC (2007). Human anti-saliva immune response following experimental exposure
to the visceral leishmaniasis vector, Lutzomyia longipalpis. Eur J Microbiol Immunol.

[13] Rohoušová I, Volf P (2006). Sand fly saliva: effects on host immune response and Leishmania
transmission. Folia Parasitol.

[14] Laurenti MD, Chaves LF, Tomokane TY, Abbasi I, Warburg A, Silveira FT (2017). Reduced Leishmania (L.) infantum chagasi parasitic loads in
humans exposed to Lutzomyia longipalpis bites in the Amazon region of
Brazil. Parasitol Open.

[15] Calzada JE, Saldaña A, González K, Rigg C, Pineda V, Santamaria AM (2015). Cutaneous Leishmaniasis in dogs: is high seroprevalence
indicative of a reservoir role?. Parasitol.

